# Production and Characterization of Fine-Grained Multielement AlCoxCrFeNi (x = 1, 0.75, 0.5) Alloys for High-Temperature Applications

**DOI:** 10.3390/ma17194897

**Published:** 2024-10-06

**Authors:** Khaja Naib Rasool Shaik, Mauro Bortolotti, Iñaki Leizaola, Miguel Angel Lagos Gomez, Cinzia Menapace

**Affiliations:** 1 Department of Industrial Engineering, University of Trento, 38122 Trento, Italy; 2 Tecnalia, E-20009 San Sebastian, Spain

**Keywords:** high entropy alloys, AlCoCrFeNi, oxidation

## Abstract

In the present work, three different AlCoxCrFeNi (x = 1, 0.75, 0.5) alloys were produced through the mechanical milling of powders and spark plasma sintering. These alloys were characterized in terms of their microstructural, mechanical, and oxidation behaviors. Mechanical milling and spark plasma sintering were chosen to achieve a fine and homogeneous microstructure. Pore-free samples were produced by properly setting the sintering parameters. The unavoidable uptake of oxygen from the powders when exposed to air after milling was advantageously used as a source of oxides, which acted as reinforcing particles in the alloy. Oxidation behavior, studied through TGA tests, showed that decreasing the Co content promotes better oxidation protection due to the formation of a dense, compact Al_2_O_3_ layer. The alloy containing the lowest amount of Co is considered a good candidate for high-temperature structural applications.

## 1. Introduction

High-entropy alloys (HEAs) are a newly developed class of materials, discovered at the beginning of the 2000s [1,2]. Compared to conventional alloys, such as Ni-based superalloys, which are characterized by the presence of a principal matrix element (Ni in this case), HEAs are composed of five or more principal elements in near-equiatomic proportions. The peculiar compositional and lattice configurational characteristics of these alloys give them very interesting properties, such as high strength even at elevated temperatures [3,4,5], and oxidation and corrosion resistance [6,7,8,9,10]. Due to these outstanding properties, HEAs show high potential for applications at elevated temperatures, mainly in aerospace components. For this purpose, they are also used as protective coatings [11,12,13]. Among the HEAs, AlCoCrFeNi is one of the most widely studied due to its very interesting properties, such as strength at high temperatures and corrosion resistance [14,15,16,17]. Moreover, the AlCoCrFeNi alloy, compared to conventional superalloys, is lighter and cheaper because of the higher content of Al and Fe relative to Ni. Ni is, in fact, one and two orders of magnitude more expensive than Al and Fe, respectively [18], and therefore, its alloys are also more costly. Regarding density, Ni superalloys have densities ranging between 8.1 and 9.4 g/cm^3^ [19], while AlCoCrFeNi has a density of around 7 g/cm^3^. Since this alloy exhibits high strength under both compression and tension up to 1000 °C [8,14], it is crucial to understand its oxidation behavior at this temperature. Moreover, most alloys designed for high-temperature applications exhibit interesting mechanical properties up to a range of 800–1000 °C, but show a significant decrease in properties above this temperature [20,21,22]. Additionally, 1000–1100 °C is considered the highest application temperature for state-of-the-art Ni-based superalloys [23,24], which serve as a reference alloy category for HEAs. Generally, regarding oxidation, HEAs demonstrate better oxidation resistance than conventional structural alloys (e.g., stainless steels, Ni-based superalloys) because they can contain higher concentrations of elements that form protective oxides, such as Cr or Al. Furthermore, HEAs, due to their sluggish diffusion kinetics [25,26], which inhibit the formation of non-protective transient oxides, exhibit higher oxidation resistance and long-term microstructural stability when exposed to high-temperature environments. There have been several investigations of the oxidation behavior of HEAs [6,10,11,12,13,27,28]. The reported results indicate that in many cases, HEAs tend to selectively oxidize, exhibiting different kinetic of oxide growth. It was specifically observed that grain size influences the oxidation behavior in alloys containing aluminum. A high number of grain boundaries facilitates the formation of an Al_2_O_3_ layer, which acts as nucleation sites for this protective oxide [29,30,31,32]. This behavior was also noted in conventional high-temperature resistant alloys, such as MCrAlY [33,34].

Regarding the alloy investigated in the present research, various authors have studied its oxidation behavior at temperatures around 1000 °C–1100 °C [9,35,36,37,38,39], highlighting its significant oxidation resistance and microstructural stability. The influence of changes in chemical compositions [9,36,37,38], as well as heat treatment [39], has been analyzed. While the major focus of prior studies has been on the development of new alloy systems, there is a lack of research on reducing high-temperature oxidation by tailoring the alloy’s microstructure, such as by reducing its grain size. Garg et al. used stationary friction processing to reduce the grain size on the surface [40] but the microstructure in the central part remained a large as-cast structure with all the typical defects of this kind of microstructure (large dendrites, chemical segregations, microporosity). The main goal of this study was to produce a series of AlCoCrFeNi alloys with a very fine, homogeneous, pore-free microstructure throughout the entire volume. Three different high-entropy alloys were produced via mechanical alloying of elemental powders and spark plasma sintering (SPS) consolidation. Thanks to these improved microstructural features, AlCoCrFeNi should exhibit interesting mechanical properties and respond well to oxidation. The three alloys produced are: the conventional AlCoCrFeNi, in which the five elements are present in equal amounts (20% atomic each), and two Co-reduced variants, where the Co content is reduced to 75% and 50%, respectively, with the reduction balanced by an increase in the other elements. Reducing Co, considered a critical raw material, is beneficial from a sustainability perspective. However, the properties of these new Co-reduced alloys must be assessed. Among these properties, oxidation behavior is particularly important since this alloy is often used in high-temperature applications.

## 2. Materials and Methods

In this study, high-purity Al, Co, Cr, Fe, and Ni elemental powders with a purity level of 99.5% (supplied from abcr GmbH, Karlsruhe, Germany) were utilized to prepare the alloys AlCoxCrFeNi (x = 1, 0.75, 0.5). The particle size of all the metal powders was <45 µm (−325 mesh). The nominal chemical composition of the three alloys is summarized in Table 1. All the elemental powders were milled in a Fritsch Pulverisette 6 ball mill (Fritsch, Idar-Oberstein, Germany) with steel balls and vials. The milling was performed at 300 rpm with a ball-to-powder weight ratio of 10:1. The milling process was conducted for 80 h, and the milling was stopped every 20 h to check the powder refinement. Ethanol was used as a process controlling agent (PCA) to prevent the cold welding of powder particles. Milling was stopped every 10 min for 20 min to avoid system overheating.

Milled powders were sintered in SPS (FCT HPD5 model S8451, FCT Systeme GmbH, Rauenstein, Germany) at 1200 °C for 2 min, applying a pressure of 50 MPa from the beginning. The initial sintering temperature, chosen on the base of previous laboratory investigations on similar alloys, was 1250 °C, but at this temperature, a liquid phase, exuding from the compact, was formed; therefore, the sintering temperature was decreased to 1200 °C. Discs of 20 mm of diameter and 6 mm of height were sintered. They were cut along the height, mounted in resin, and prepared for metallographic analysis.

Oxidation tests were conducted in a Thermo Gravimetric Analyzer (TGA-STA 409 Luxx, Netzsch, Selb, Germany) at 1000 °C for 10 h under a flux of air, recording the mass increase. Beforehand, each test sample’s surface was measured. Oxidation time was limited to 10 h, since that is sufficiently long enough to study the oxidation kinetic (i.e., the shape of the oxidation curve) [41,42,43].

Scanning electron microscope (SEM) analysis was used to examine the sintered and oxidized samples using a JEOL IT300 scanning electron microscope (JEOL Ltd., Akishima, Japan) at 20 keV voltage, equipped with an Brunker energy-dispersive X-ray spectroscope (EDXS) (Bruker, Billerica, MA, USA) and with an Xflash 630M detector (Bruker, Billerica, MA, USA) attached.

XRD data were collected on a Rigaku DMAX III diffractometer equipped with a Cu anode source operating at 40 kV and 30 mA with a graphite monochromator on the secondary beam. Samples were scanned in reflection geometry (omega-theta) on the 20°–120° interval, with a 0.02° scan resolution and 2 s counting time per step.

The Vickers hardness measurements were performed on a Vickers microhardness tester (FM-310, FUTURE-TECH CORP, Kawasaki Kanagawa, Japan). The measurements were taken with 100 g of load with 10 s of dwell time. A total of 10 measurements were taken for each sample.

Applying a load of 10 kg, Vickers indentations were used to calculate fracture toughness K_IC_ through the Equation (1) proposed by Shubert et al. that refers to the Palmqvist method [44]:

(1) $$K_{IC}=0.0028\;\cdot \;\sqrt{H}\;\cdot \sqrt{\frac{P}{\sum l_{i}}}$$

where *H* = Vickers hardness in N/mm^2^, *P* = load in N, and *l_i_* = crack lengths in mm.

## 3. Results and Discussion

### 3.1. Milled Powders

**Figure 1:**
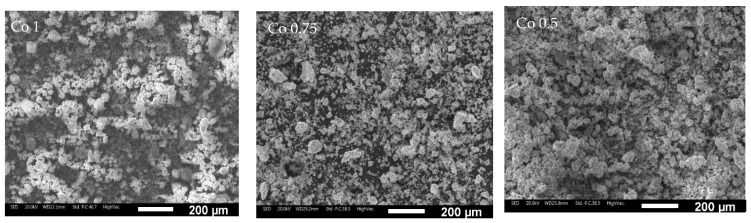
After 80 h, milled powders of the three alloys (Co1, Co0.75, Co0.50).

**Figure 2:**
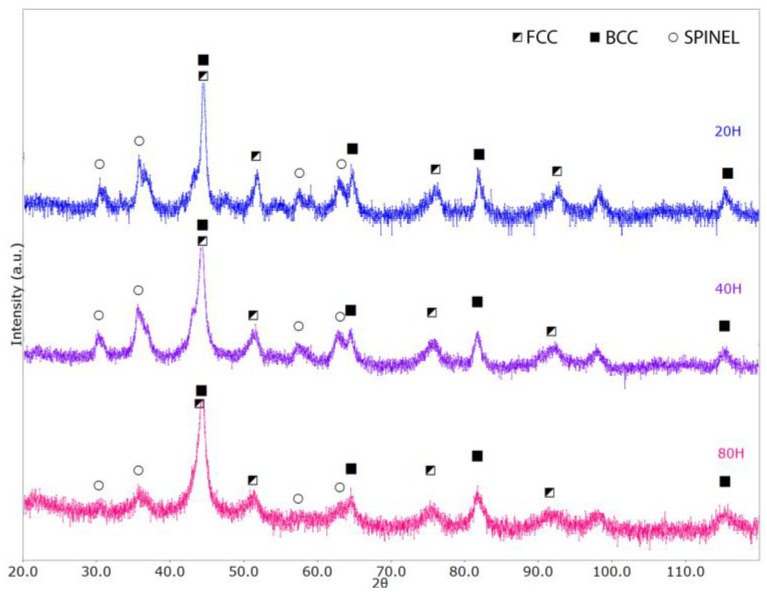
XRD spectra of the Co1 powder milled for 20, 40, and 80 h.

The morphology of AlCoxCrFeNi (x = 1, 0.75, 0.5) HEA powders after 80 h of milling is shown in Figure 1. The particle size and morphology are similar for the three alloys. Due to the heavy collision of the milling balls against the powder particles, continuous deformation, welding, and fragmentation occur, promoting the mechanical alloying process thanks to the elements’ inter-diffusivity [45,46]. XRD analysis of the Co1 milled powders after 20, 40, and 80 h of milling is shown in Figure 2. The analysis indicates the presence of three phases: an FCC, a BCC, and a spinel-like phase, the last indicating the formation of oxides when the milled powder is exposed to air. Milling time was stopped after 80 h, since no further powder evolution was occurring.

### 3.2. Sintered Samples

**Figure 3:**
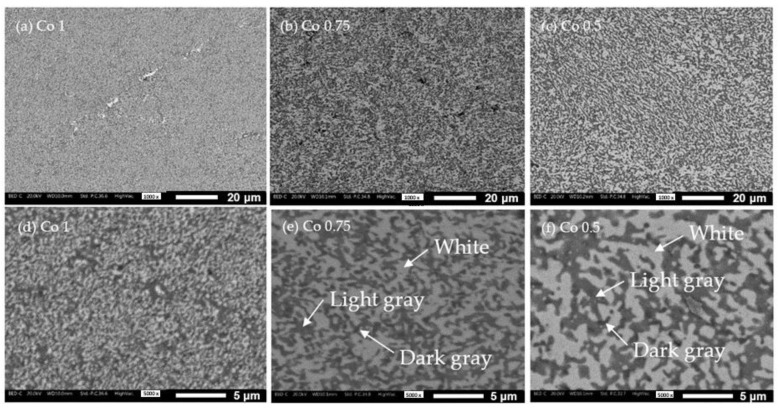
Microstructures of SPSed alloys Co1, Co0.75, and Co0.50 at 1000× (**a**–**c**) and at 5000× (**d**–**f**).

Figure 3 shows the microstructures of the three alloys observed under the SEM at two different magnifications (1000× and 5000×). Full density samples were obtained at this sintering temperature. The microstructure of the three alloys is very fine and homogeneous, thanks to the prolonged milling process and rapid sintering. The finest grain size is observed in Co1, and as the Co content decreases, the grain size increases.

**Figure 4:**
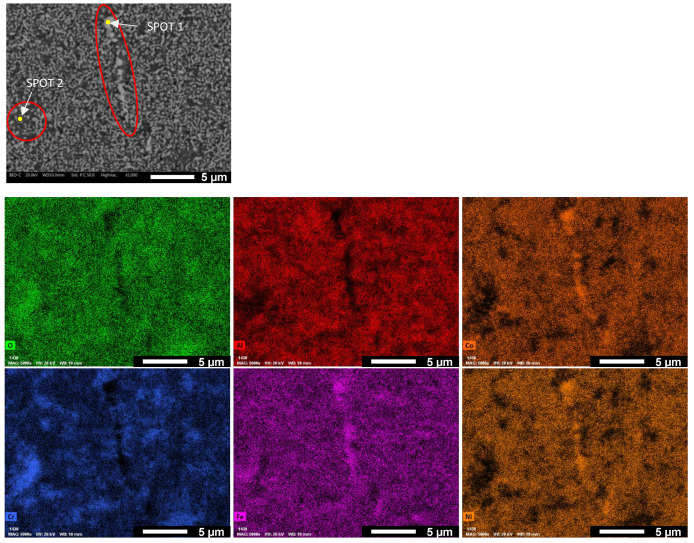
EDXS map of Co1 sample.

Three different phases are recognizable by their distinct gray-scale colors in back-scattered mode analysis (BSE) at SEM: white, light gray, and dark gray, indicated by arrows on the micrographs. To investigate the composition of the three phases, EDXS maps were collected on the three samples. An example is shown in Figure 4, related to Co1. The map reveals that the white areas correspond to Co-Fe-Ni rich regions. Spot analysis (Table 2) indicates the presence of Al and Cr in these areas. This phase is clearly identified by the white stripe in the center of the micrograph. Conversely, the black area, circled in the micrograph, is rich in O, Cr, and partially Fe, indicating it is a mixed oxide. The light gray areas are so small and uniformly distributed that they could not be clearly distinguished and analyzed.

The sintering temperature of 1200 °C was chosen because sintering the Co1 powder at a higher temperature (1250 °C) resulted in some liquid phase formation and residual porosity, leading to a density of 5.92 g/cm^3^. Additionally, during sintering at 1250 °C, liquid exudation from the dye was observed, which can cause breakage of the punches and dye. The densities of the three alloys, measured using the Archimedes method, are 6.11 g/cm^3^, 6.13 g/cm^3^, and 6.24 g/cm^3^ for Co1, Co0.75, and Co0.50, respectively. These density values are lower than those found in the literature due to the presence of a significant amount of oxides. Despite the low density values, all the metallographic sections examined (in both longitudinal and transversal directions) confirmed the absence of porosity. This microstructure is much finer, more homogeneous, and defect-free compared to those typically obtained through conventional casting techniques, the sintering of not heavily milled powders, or sintering in the presence of a liquid phase [17,47,48,49].

**Figure 5:**
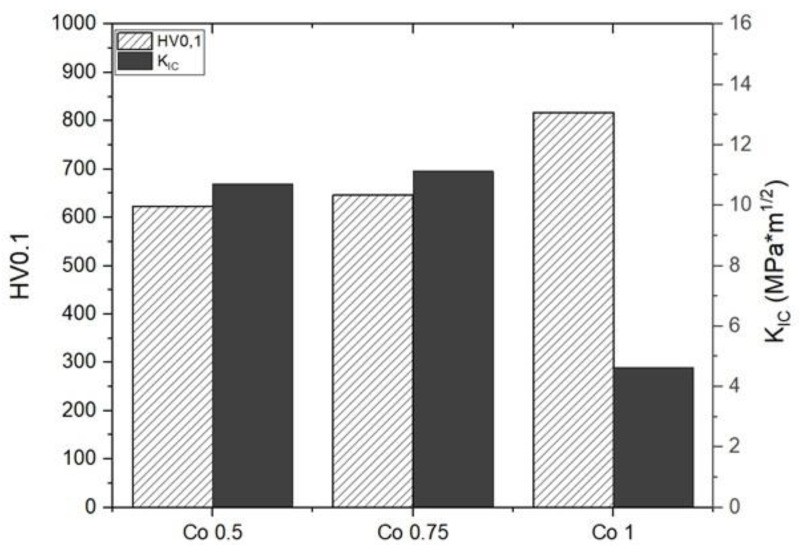
Vickers microhardness HV0.1 and K_IC_.

The Vickers microhardness measured on AlCoxCrFeNi SPSed HEAs is shown in Figure 5. Co1 has a higher microhardness due to its finer microstructure respect to the other two compositions. In previous investigations [50,51], microhardness values were calculated for the same alloy sintered through self-propagating-high-temperature synthesis (SHS) and suction cast method. The microhardness values for the SHS method were in the range of 380–510 HV, and for the suction cast method between 520 and 530 HV, varying the process parameters and the molar ratios of the constituents. A bigger grain size and a more inhomogeneous phase distribution is the reason of the lower microhardness of these materials with respect to the ones investigated in the present research. Moreover, in the present investigation, the consistent amount of oxides formed by exposing the powder to air after milling contributes to the hardness increase. As observed in a previous paper by the same authors [52], the oxide particle reinforcement leads to high hardness, kept also at high temperature, thanks to the oxides’ stability.

**Figure 6:**
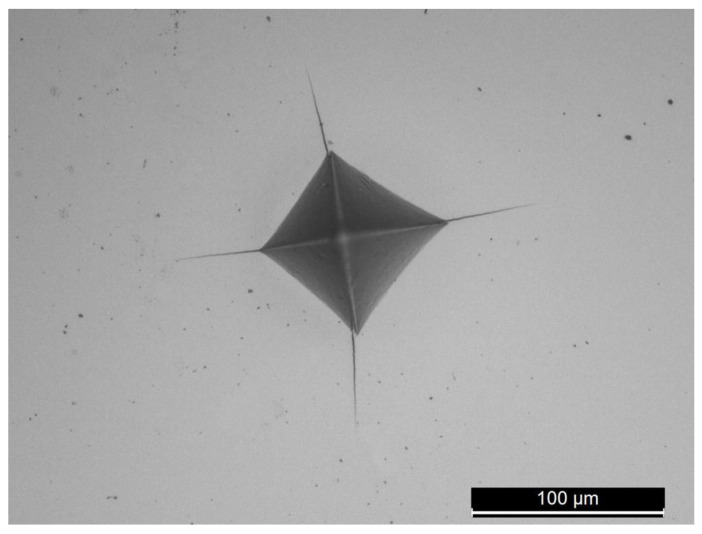
Vickers indentation showing cracks at corners in sample Co1.

K_IC_ values measured through Palmqvist method are reported in Figure 5, along with a hardness of HV0.1. An example of hardness indentation with cracks starting at the corners of the imprint, used for the calculation of K_IC_, is shown in Figure 6. K_IC_ is much lower in the Co1 sample due to its higher hardness, which induces brittle behavior in the material. The fracture toughness of these alloys is in the typical range for BCC HEAs (0.2–20 MPa m^1/2^), while FCC exhibit much higher values (up to 250 MPa m^1/2^) [47]. This is attributed to the presence of oxide particles in the microstructure, which makes these alloys similar to fine-grained metal matrix composites. As discussed in a previous paper, the oxygen uptake by the milled powders has a beneficial effect. This uptake can be advantageously transformed to strengthen the matrix and improve its thermal stability.

**Figure 7:**
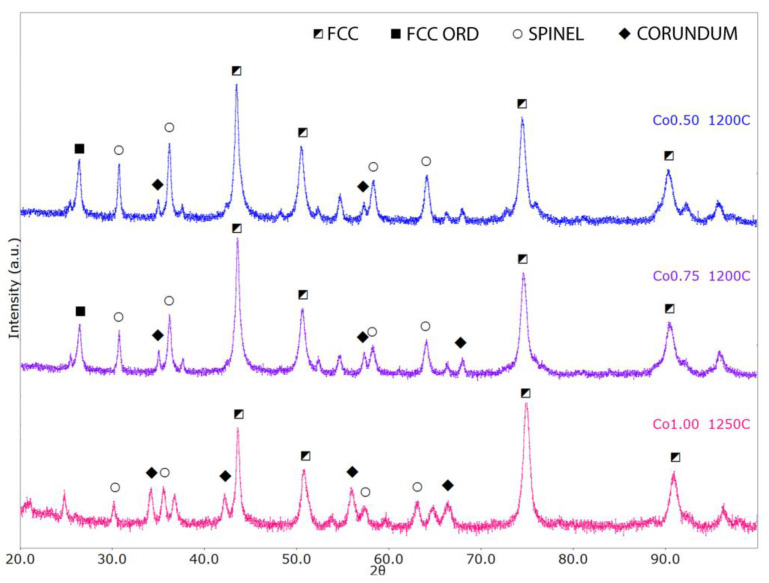
Rietveld fit of XRD data collected on Co 0.50, Co 0.75, and Co1.

The alloys phase composition was investigated also through XRD analysis. In all samples, three main crystallographic phases were identified, namely an FCC structure, a spinel-like structure (likely representing an oxide of the form M_3_O_4_), and an Al_2_O_3_-like structure (mainly alumina or other mixed oxides having the same crystal structure). These phases can be correlated to the three phases observed on the metallographic samples (white, light gray, and dark gray). Quantitative phase analysis was conducted by means of ReX software (Version v.0.9.4.) [53], refining phases’ weight fractions as well as their lattice parameters and significant microstructural parameters. Numerical results are reported in Table 3, while final fitting plots are shown in Figure 7.

Both the FCC and spinel phases weight fractions significantly decrease with the higher Co amount, with the Al_2_O_3_ phase showing the opposite trend. In the samples 0.5Co and 0.75Co, an additional diffraction peak is observed at approximately 26.5° 2θ; this is possibly a superlattice reflection due to the presence of additional ordering of the FCC lattice, though this feature was not quantitatively modeled (FCC ORD). Microstructural parameters are relatively stable, with only a noticeable decrease in the average crystallite sizes for the Al_2_O_3_ phase with an increasing Co content. The presence of a high amount of oxides is attributed to the intense oxidation of the powders when they are exposed to air after the milling process, due to their very fine particle size and chemical composition.

In all cases, the exact chemistry of the crystallographic phases cannot be quantitatively determined in a reliable manner by means of diffraction data alone. Though, given the significant differences in lattice parameters with the reference literature’s structures, as well as among the different samples, some semiquantitative trends can be described. In particular:

- With an increase in the Co content, the FCC lattice exhibits lattice/cell volume shrinking, whereas the two oxides show the opposite behavior. In general, we can expect larger unit cell volumes (with respect to the reference structures) to be associated with substitutional disorder, whereas smaller volumes can be possibly associated with particular chemical element segregation.
- The FCC crystal structure presents a decrease in the lattice parameter with increasing Co content, with values varying between 3.60 Å and 3.58 Å. For reference, Ni, Co, and g-Fe standard FCC lattice parameters are, respectively, 3.53 Å, 3.55 Å, and 3.59 Å.
- The spinel cubic lattice exhibits a quite significant lattice parameter increase from 8.21 Å to 8.32 Å with increasing Co content, with reference structures like Co_3_O_4_ and Fe_3_O_4_ having lattice parameters equal to 8.08 Å to 8.39 Å, respectively. This could also be related to the Al-depletion in the matrix consequent to the Al_2_O_3_ phase increase.
- For the Al_2_O_3_-like lattice, we have lattice parameters varying between 4.78 Å and 4.87 Å (a) and between 13.03 Å and 13.28 Å (c); for comparison, the reference Al_2_O_3_ and Cr_2_O_3_ structures have lattice parameters values equal to a = 4.76 Å, c = 12.99 Å and a = 4.96 Å, c = 13.6 Å, respectively. The α-Al_2_O_3_ phase was also observed by Wang et al. [17], who obtained the alloy via mechanical milling and SPS. This oxide shows high chemical stability and gives oxidation resistance.

### 3.3. Oxidation Behavior

**Figure 8:**
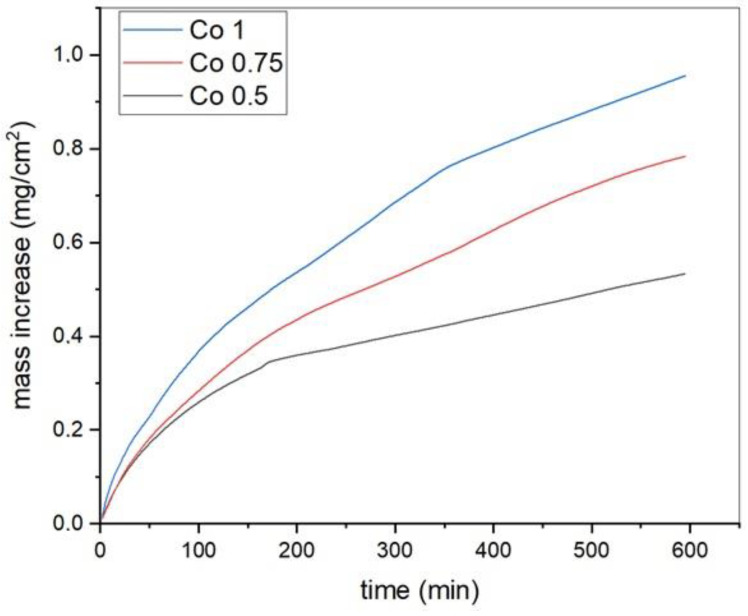
Oxidation curves of Co1, Co0.75, and Co0.50.

Oxidation curves obtained through thermogravimetric analysis are shown in Figure 8. These curves exhibit a parabolic oxidation kinetic, which typically occur when oxidation is controlled by the ionic diffusion of oxidizing species. The parabolic oxide growth rate constants (kp) derived from these curves using Equation (2) are reported in Table 3 [41]. They were calculated from the slopes of (ΔW/A)^2^ versus t plots for each alloy, where W represents the weight increase and A represents the sample surface area.

X^2^ = kp × t (2)

where X is the weight increase/surface, and t is the time.

As shown in Table 4, the constant values kp decrease with decreasing amounts of cobalt, indicating higher oxidation resistance when the amount of Co is lower. In this case, the amounts of other elements in the alloy, particularly Al and Cr, are higher, which form protective oxides. These data are consistent with the oxidation tests performed by Butler et al., who measured a weight increase of 0.8 mg/cm^2^ after 10 h at 1050 °C for the standard alloy AlCoCrFeNi, in equiatomic percentage (Co1 in the present investigation), produced via arc melting [9].

**Figure 9:**
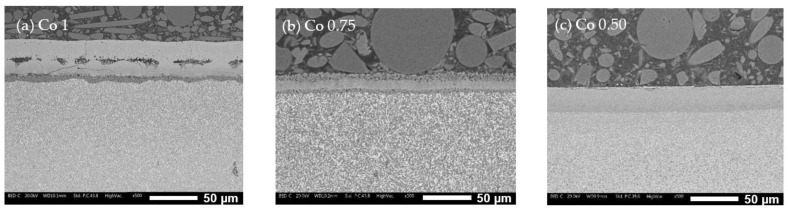
Oxide layers formed on Co1 (**a**), Co0.75 (**b**), and Co 0.50 (**c**) alloy after 10 h at 1000 °C in air.

**Figure 10:**
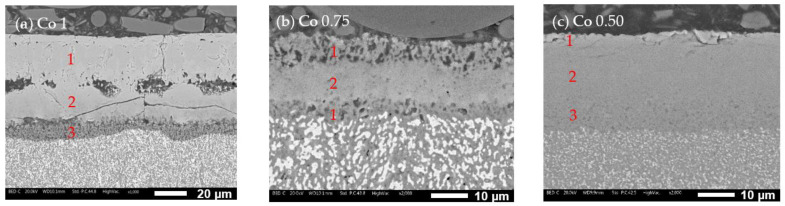
Oxide layers formed on Co1 (**a**), Co0.75 (**b**), and Co0.50 (**c**) observed under the SEM at higher magnification.

The oxide layer investigation through SEM analysis shows varying thicknesses and compositions for the oxide layers formed during oxidation tests. Figure 9 and Figure 10 illustrate these layers at two different magnifications for the three alloys (a = Co1, b = Co0.75, and c = Co0.50). The oxide layers exhibit different thicknesses, with the mean values obtained from 20 measurements being 47 µm for Co1, 28 µm for Co0.75, and 34 µm for Co0.50. The characteristics of the oxidation layers also differ significantly:

- Co1 (Figure 10a): The oxide layer appears to be divided into three parts. The outermost layer (layer 1) is characterized by grain-like structures. Below this, separated by poor bonding with many longitudinal open cracks, is a second layer (layer 2) with no visible grains and numerous cracks, indicating brittleness. The third layer (layer 3), in contact with the alloy surface, is much thinner and shows some microporosity.
- Co0.75 (Figure 10b): This alloy features a “sandwich” layer structure, with the top and bottom parts exhibiting similar microporosity (layers 1), while the middle layer (layer 2) is more compact.
- Co0.50 (Figure 10c): This alloy also has three layers: an external very thin layer with various cracks (layer 1), a thicker compact internal layer in the middle (layer 2), and a bottom layer similar to layer 2 but with diffused microporosity (layer 3).

**Figure 11:**
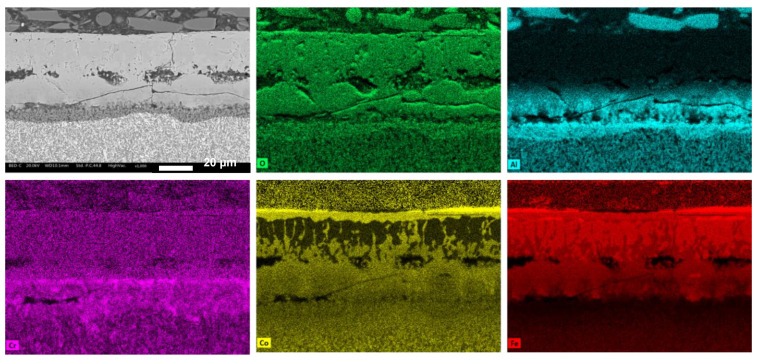
EDXS map of the oxide layer formed on Co1 alloy.

**Figure 12:**
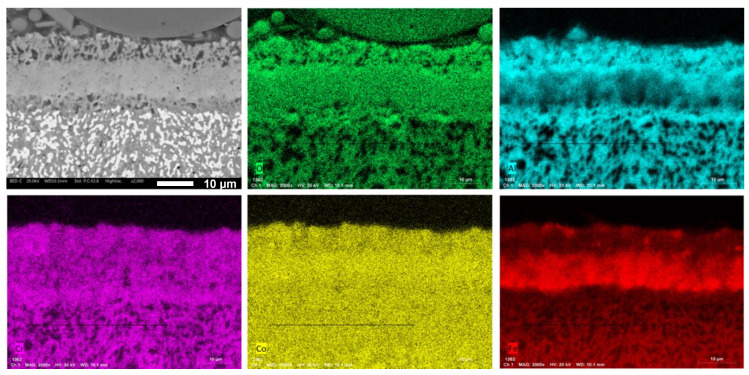
EDXS map of the oxide layer formed on Co0.75 alloy.

**Figure 13:**
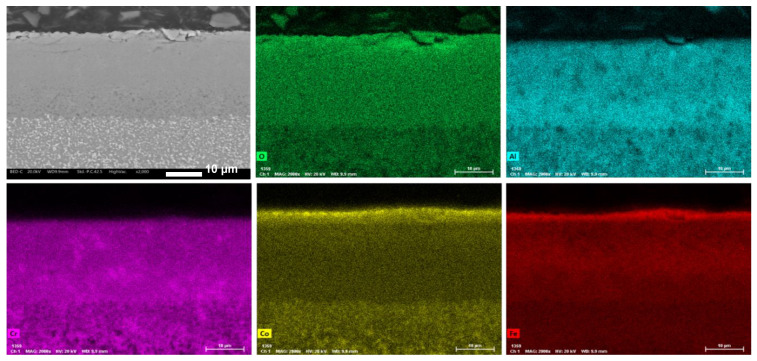
EDXS map of the oxide layer formed on Co0.50 alloy.

EDXS maps of the three different oxide layers are shown in Figure 11, Figure 12 and Figure 13, providing insights into the composition of these layers. In the Co1 sample, the external layer (layer 1) consists of Co and Fe oxides, which are not protective oxides. Layer 2 is composed of Al oxide, and layer 3 contains both Al and Cr oxides.

For Co0.75, the two outer layers (layer 1) are made of porous Al_2_O_3_ and Cr_2_O_3_, while the central layer (layer 2) consists of Fe and Co oxides.

In the Co0.50 sample, the very thin outer layer (layer 1) is composed of Fe and Co oxides. Beneath this is an extremely compact layer (layer 2) made of protective Al_2_O_3_ and Cr_2_O_3_ oxides. The bottom layer (layer 3) in this material is composed of porous alumina.

The presence of a thick and dense layer of Al_2_O_3_ and Cr_2_O_3_, along with an underlayer of Al_2_O_3_, accounts for the higher oxidation resistance of the Co0.50 alloy, benefiting from its higher Al and Cr content. Moreover, only this alloy shows a very compact oxide layer. In Co1, the thicker oxide layer and its composition created interlayer cracks due to thermal stresses and the formation of interfacial voids. In Co0.75, the Al_2_O_3_ and Cr_2_O_3_ layers are porous, and the compact layer is composed of the less protective Fe and Co oxides. Only Co0.50 has a dense and compact Al_2_O_3_ layer. The presence of a higher amount of Al_2_O_3_ in the bottom part of the oxide layer suggests that the growth of the Al_2_O_3_ scale is dominated by inward O diffusion. Despite the fine grain size, which promotes the formation of Al_2_O_3_, in the Co1 sample, the presence of a significant amount of this oxide already in the microstructure before the oxidation test (as reported in Table 3) reduces the amount of Al available to form a protective alumina layer. Therefore, Co1 has the lowest oxidation resistance. A similar oxidation behavior is observed in alumina-forming austenitic stainless steels and Al-rich Ni superalloys [54,55], which are considered references for HEAs in high-temperature applications. In these conventional alloys, the oxidation behavior is characterized by the formation of a protective underlayer of Al_2_O_3_, providing high oxidation resistance up to approximately 900 °C for stainless steels, and even higher temperatures for superalloys. Many Ni-Al-Cr alloys gives oxidation constants kp in the range 10^−11^–10^−12^ g^2^·cm^−4^ s^−1^, which are similar to the ones found in the present investigation [54].

In light of the previous results, Co0.50 is the most interesting alloy among those tested in the present investigation, as it shows a good combination of a fine-grained matrix (with lattice distortion confirmed by XRD), pore-free microstructure, and the correct amount of oxide particles, homogeneously distributed within the matrix. These characteristics provide this alloy with a good combination of hardness (strength), K_IC_, and oxidation resistance.

## 4. Conclusions

Three multielement alloys, AlCoxCrFeNi (x = 1, 0.75, 0.5), have been produced via mechanical alloying and spark plasma sintering (SPS) consolidation. The cobalt content was decreased and replaced by increasing the content of the other elements, allowing for a comparison between the new alloys and the full cobalt alloy. A prolonged milling time, coupled with SPS consolidation, was chosen to achieve a very fine, homogeneous, pore-free microstructure reinforced by oxides. The oxidation behavior of these alloys, analyzed through TGA curves, shows a parabolic kinetic with the formation of oxide multi-layers. The best oxidation resistance is observed in the Co0.50 alloy, as the reduction of Co in this alloy is accompanied by a higher amount of Al, which, due to the fine grain size, rapidly forms a dense protective Al_2_O_3_ scale.

## Figures and Tables

**Table 1:** Composition of the three high entropy alloys.

Alloys	Nominal Composition (at%)					
	LABEL	Al	Co	Cr	Fe	Ni
AlCoCrFeNi	Co1	20	20	20	20	20
AlCo0.75CrFeNi	Co0.75	21.25	15	21.25	21.25	21.25
AlCo0.5CrFeNi	Co0.50	22.5	10	22.5	22.5	22.5

**Table 2:** EDS analysis on SPOT 1 e SPOT 2 of Figure 4.

Alloys	Nominal Composition (wt%)					
	O	Al	Co	Cr	Fe	Ni
SPOT 1	1.04	2.94	30.16	10.00	26.67	29.19
SPOT 2	7.63	7.67	15.18	28.66	27.56	13.29

**Table 3:** Quantitative results obtained from XRD data modeling.

Phase/Constituent		Co0.50_1200C	Co0.75_1200C	Co1.00_1250C
	Rwp (weighted profile R-factor)	0.2838	0.2708	0.2593
FCC	Weight fraction %	49.70%	52.61%	21.13%
	Domain size [Å]	179	192	196
	Lattice strain (r.m.s.)	2.97 × 10^−3^	2.96 × 10^−3^	2.90 × 10^−3^
	Cell a [Å]	3.600	3.598	3.579
Spinel	Weight fraction %	41.22%	28.99%	10.06%
	Domain size [Å]	261	280	214
	Cell a [Å]	8.215	8.228	8.324
Al_2_O_3_	Weight fraction %	9.07%	18.39%	68.81%
	Domain size [Å]	595	466	277
	Cell a [Å]	4.780	4.781	4.868
	Cell c [Å]	13.033	13.035	13.279

**Table 4:** Oxidation constants kp.

	Kp (g^2^·cm^−4^·s^−1^)
Co 1	26.83 × 10^−12^
Co 0.75	17.82 × 10^−12^
Co 0.50	7.43 × 10^−12^

## Data Availability

The data presented in this study are available on request from the corresponding author.
